# Supplementary material for: Computational Approach to Drug Penetration across the Blood-Brain and Blood-Milk Barrier Using Chromatographic Descriptors
Source: Cells. 2023 Jan 27;12(3):421. doi: 10.3390/cells12030421 (PMC9913351; doi:10.3390/cells12030421)

**Table S1.** Biological properties of 37 APIs

| API | B1 | B2 | B3 | $BB_{vivo}$ | CNS+/- | LactMed | LLL H | log U/D | M/P | PB | PhCharge |
|---|---|---|---|---|---|---|---|---|---|---|---|
| **acetazolamid** | -1.60 | -1.29 | 0.52 | | 1 | 1 | 2 | -0.24 | 0.28 | 0.98 | -1 |
| **amitriptyline** | 0.76 | 0.50 | -0.45 | 0.89 | 1 | 1 | 2 | -1.98 | 0.92 | 0.95 | 1 |
| **bupivacaine** | 0.16 | 0.03 | 1.25 | | 1 | 1 | 2 | -0.93 | 0.37 | 0.95 | 1 |
| **chloroquine** | 0.39 | 0.10 | 0.89 | | 1 | 1 | 2 | -3.27 | 2.65 | 0.55 | 2 |
| **citalopram** | 0.13 | -0.03 | -0.23 | | 1 | 2 | 2 | -2.37 | 1.03 | 0.80 | 1 |
| **clomipramine** | 0.79 | 0.44 | -0.28 | | 1 | 1 | 2 | -2.26 | 1.13 | 0.98 | 1 |
| **colchicine** | -0.93 | -0.78 | -1.99 | | 1 | 2 | 4 | | 0.95 | 0.39 | 0 |
| **diltiazem** | -0.02 | -0.40 | -1.22 | | 1 | 1 | 3 | -1.74 | 0.99 | 0.75 | 1 |
| **doxepin** | 0.54 | 0.35 | -0.18 | | 1 | 1 | 5 | -2.20 | 1.37 | 0.76 | 1 |
| **duloxetine** | 0.56 | 0.21 | -0.65 | | 1 | 1 | 3 | -2.82 | 0.78 | 0.90 | 1 |
| **eletriptan** | -0.19 | -0.30 | -0.84 | | 1 | 1 | 3 | -3.15 | 0.25 | 0.85 | 1 |
| **ethambutol** | -0.86 | -0.49 | -0.52 | | 0 | 1 | 2 | -2.39 | 1.00 | 0.25 | 1 |
| **fluvoxamine** | -0.14 | -0.36 | 0.68 | | 1 | 1 | 2 | -2.19 | 1.33 | 0.79 | 1 |
| **gabapentin** | -0.63 | -0.47 | -0.19 | | 1 | 1 | 2 | -0.29 | 1.00 | 0.03 | 0 |
| **gentamycin** | -3.13 | -2.65 | -2.40 | | 0 | 1 | 2 | -2.57 | 0.36 | 0.15 | 5 |
| **hydrochlorotiazide** | -1.62 | -1.35 | -2.29 | | 0 | 1 | 2 | -1.75 | 0.38 | 0.68 | 0 |
| **itraconazole** | -0.66 | -1.07 | 3.68 | | 0 | 1 | 2 | -0.73 | 1.14 | 1.00 | 0 |
| **ketorolac** | -0.33 | -0.40 | 0.23 | | 1 | 1 | 2 | -2.91 | 0.03 | 0.99 | -1 |
| **levetiracetam** | -0.93 | -0.47 | -1.02 | | 1 | 1 | 3 | | 1.00 | 0.10 | 0 |
| **lincomycin** | -1.62 | -1.47 | -0.43 | | 0 | | 2 | -1.58 | 0.15 | 0.70 | 1 |
| **mesalazine** | -0.99 | -0.79 | -1.55 | | 0 | 1 | 3 | -5.30 | 2.69 | 0.43 | -1 |
| **minoxidil** | -1.00 | -0.95 | -0.18 | | 1 | | 3 | -1.66 | 0.82 | 0.00 | 0 |
| **nitrendipine** | -0.92 | -1.22 | -1.09 | | 0 | 1 | 2 | -4.41 | 0.30 | 0.99 | 0 |
| **ofloxacin** | -0.66 | -0.63 | -1.97 | | 0 | 1 | 2 | -2.01 | 1.26 | 0.32 | -1 |
| **oxcarbamazepine** | -0.55 | -0.47 | -0.87 | | 1 | | 3 | -6.53 | 0.50 | 0.40 | 0 |
| **pefloxacin** | -0.52 | -0.48 | -1.44 | | 0 | | | -7.04 | 0.96 | 0.25 | -1 |

| | | | | | | | | | | | |
|---|---|---|---|---|---|---|---|---|---|---|---|
| **pregabalin** | -0.63 | -0.47 | -0.22 | | 1 | 1 | 3 | -0.57 | 0.55 | 0.00 | 0 |
| **primidone** | -0.60 | -0.38 | -0.31 | | 1 | 2 | 3 | -5.06 | 0.72 | 0.70 | 0 |
| **propylthiouracil** | -0.29 | -0.11 | -0.31 | | 1 | 1 | 2 | -0.43 | 0.17 | 0.82 | 0 |
| **quinine** | -0.11 | -0.18 | -0.07 | | 1 | 1 | 2 | -2.08 | 0.23 | 0.70 | 1 |
| **rifampicin** | -2.81 | -2.98 | -0.67 | | 0 | 1 | 2 | -2.24 | 0.40 | 0.89 | 1 |
| **rimantadine** | 0.26 | 0.13 | 0.43 | | 1 | | 3 | -3.56 | 2.00 | 0.40 | 1 |
| **timolol** | -0.85 | -0.73 | -0.06 | | 0 | 1 | 2 | -2.15 | 0.80 | 0.10 | 1 |
| **tinidazole** | -1.35 | -1.02 | -0.65 | | 1 | 1 | 3 | -4.90 | 1.14 | 0.12 | 0 |
| **tramadol** | 0.01 | 0.02 | -0.67 | | 1 | 1 | 2 | -2.41 | 2.30 | 0.20 | 1 |
| **trimethoprim** | -1.33 | -1.14 | -1.90 | | 1 | 1 | 2 | -0.30 | 1.25 | 0.44 | 1 |
| **warfarin** | -0.33 | -0.47 | -0.44 | | 1 | 0 | 2 | -2.70 | 0.00 | 0.99 | -1 |

**Table S2.** Physicochemical properties of 37 APIs

| API | a/b/n code[1] | DM | eH | eH-eL | eL[2] | HA | HD | log D | log P | MW[3] | pKa | PSA[4] | Sa[3] | V[3] |
|---|---|---|---|---|---|---|---|---|---|---|---|---|---|---|
| **acetazolamid** | 0 | 0.89 | -9.06 | -9.13 | 0.69 | 5 | 2 | -0.55 | -0.26 | 2.22 | 7.44 | 11.50 | 4.69 | 2.9 |
| **amitriptyline** | 1 | 6.42 | -9.9 | -8.20 | -17.01 | 1 | 0 | 3.15 | 4.41 | 2.77 | 9.18 | 0.32 | 3.61 | 1.56 |
| **bupivacaine** | 0 | 3.02 | -9.03 | -9.37 | 3.37 | 2 | 1 | 2.80 | 3.31 | 2.88 | 8.13 | 3.23 | 4.78 | 3.02 |
| **chloroquine** | 1 | 4.4 | -8.6 | -8.03 | -5.71 | 3 | 1 | 1.87 | 4.41 | 3.20 | 10.47 | 2.82 | 5.94 | 3.11 |
| **citalopram** | 1 | 2.83 | -7.98 | -8.86 | 8.83 | 3 | 0 | 0.39 | 3.48 | 3.24 | 9.57 | 3.63 | 5.88 | 3.14 |
| **clomipramine** | 1 | 1.53 | -8.49 | -8.65 | 1.56 | 2 | 0 | 3.50 | 4.94 | 3.15 | 9.46 | 0.65 | 5.11 | 3.05 |
| **colchicine** | 0 | 5.95 | -9.11 | -8.52 | -5.91 | 6 | 1 | 0.92 | 1.07 | 3.99 |  | 8.31 | 5.47 | 3.61 |
| **diltiazem** | 1 | 2.43 | -8.71 | -8.52 | -1.86 | 4 | 0 | 2.10 | 4.73 | 4.15 | 8.94 | 5.91 | 6.17 | 3.96 |
| **doxepin** | 1 | 0.31 | -9.11 | -9.03 | -0.81 | 2 | 0 | 2.08 | 3.84 | 2.79 | 9.40 | 1.25 | 4.69 | 2.81 |
| **duloxetine** | 1 | 0.96 | -9.01 | -8.63 | -3.82 | 2 | 1 | 1.24 | 4.81 | 2.97 | 10.02 | 2.13 | 4.56 | 2.84 |
| **eletriptan** | 1 | 4.88 | -8.5 | -7.90 | -5.96 | 3 | 1 | 0.36 | 2.98 | 3.83 | 10.35 | 5.32 | 4.69 | 3.57 |
| **ethambutol** | 1 | 1.11 | -9.11 | -11.45 | 23.39 | 4 | 4 | -2.21 | -0.29 | 2.04 | 9.59 | 6.45 | 4.95 | 2.18 |
| **fluvoxamine** | 1 | 3.9 | -9.69 | -8.87 | -8.25 | 4 | 1 | 1.15 | 3.71 | 3.18 | 9.39 | 5.68 | 5.89 | 2.79 |
| **gabapentin** | 0 | 1.56 | -9.35 | -10.19 | 8.36 | 3 | 2 | -1.31 | 1.08 | 1.71 | 7.50 | 6.33 | 2.38 | 1.7 |
| **gentamycin** | 1 | 4.31 | -9.34 | -11.20 | 18.62 | 12 | 8 | -7.81 | -2.04 | 4.78 | 9.77 | 19.97 | 4.71 | 4.25 |
| **hydrochlorotiazide** | 0 | 7.27 | -9.64 | -8.46 | -11.83 | 5 | 3 | -0.09 | -0.02 | 2.98 | 8.95 | 11.84 | 3.85 | 2.01 |
| **itraconazole** | 0 | 6.11 | -8.42 | -7.65 | -7.7 | 9 | 0 | 4.26 | 4.53 | 6.73 | 6.47 | 10.08 | 8.43 | 6.15 |
| **ketorolac** | -1 | 2.14 | -9.19 | -8.83 | -3.63 | 3 | 1 | -0.95 | 2.68 | 2.55 | 4.29 | 5.93 | 3.87 | 2.39 |
| **levetiracetam** | 0 | 5.71 | -9.8 | -10.45 | 6.49 | 2 | 1 | -0.67 | -0.88 | 1.70 |  | 6.34 | 3.09 | 1.62 |
| **lincomycin** | 1 | 3.92 | -8.5 | -8.50 | 0.04 | 8 | 5 | -0.48 | 0.72 | 4.07 | 8.78 | 12.60 | 6.35 | 3.89 |
| **mesalazine** | -1 | 3.8 | -8.18 | -8.06 | -1.23 | 4 | 3 | -2.61 | 0.74 | 1.53 | 1.90 | 8.36 | 2.49 | 1.28 |
| **minoxidil** | 0 | 4.2 | -8.07 | -8.10 | 0.29 | 5 | 2 | 0.65 | 1.62 | 2.09 | 5.54 | 9.36 | 3.16 | 1.96 |
| **nitrendipine** | 0 | 6.92 | -9.09 | -8.00 | -10.94 | 5 | 1 | 3.50 | 3.81 | 3.60 | 2.79 | 11.05 | 5.3 | 2.98 |
| **ofloxacin** | -1 | 4.68 | -8.72 | -7.82 | -8.97 | 7 | 1 | -0.65 | 1.85 | 3.61 | 5.19 | 7.33 | 4.74 | 3.07 |
| **oxcarbamazepine** | 0 | 3.46 | -9.31 | -8.84 | -4.73 | 2 | 1 | 1.25 | 1.66 | 2.52 | 13.73 | 6.34 | 3.02 | 2.26 |
| **pefloxacin** | -1 | 9.41 | -9.01 | -7.97 | -10.45 | 6 | 1 | 0.52 | 1.92 | 3.33 | 0.16 | 6.41 | 4.66 | 2.91 |
| **pregabalin** | 0 | 1.67 | -9.53 | -10.46 | 9.29 | 3 | 2 | -1.38 | 1.09 | 1.59 | 7.77 | 6.33 | 3.54 | 1.65 |
| **primidone** | 0 | 2.84 | -9.78 | -9.77 | -0.15 | 2 | 2 | 0.40 | 0.83 | 2.18 | 12.26 | 5.82 | 3.19 | 2.02 |

| | | | | | | | | | | | | | | |
|---|---|---|---|---|---|---|---|---|---|---|---|---|---|---|
| **propylthiouracil** | 0 | 5.57 | -9.2 | -7.93 | -12.71 | 1 | 2 | 1.18 | 1.15 | 1.70 | 7.63 | 4.11 | 3.32 | 1.51 |
| **quinine** | 1 | 1.73 | -9.55 | -9.54 | -0.14 | 4 | 1 | 1.58 | 2.82 | 3.24 | 9.28 | 4.56 | 4.22 | 3.06 |
| **rifampicin** | -1 | | | | | 14 | 6 | -0.46 | 2.05 | 8.23 | 4.96 | 22.02 | | |
| **rimantadine** | 1 | 1.22 | -9.33 | -12.28 | 29.54 | 1 | 1 | 0.08 | 3.31 | 1.79 | 10.76 | 2.60 | 2.43 | 1.91 |
| **timolol** | 1 | 3.06 | -9.17 | -8.05 | -11.24 | 7 | 2 | -1.39 | 1.28 | 3.16 | 9.35 | 7.97 | 4.95 | 2.88 |
| **tinidazole** | 0 | 2.34 | -10.52 | -9.24 | -12.82 | 5 | 0 | -0.27 | -0.29 | 2.47 | 2.30 | 9.78 | 4.41 | 2 |
| **tramadol** | 1 | | | | | 3 | 1 | 0.36 | 2.32 | 2.63 | 9.61 | 3.27 | | |
| **trimethoprim** | 0 | 2.09 | -8.8 | -8.68 | -1.22 | 7 | 2 | 0.58 | 0.59 | 2.90 | 6.90 | 10.55 | 4.14 | 2.63 |
| **warfarin** | -1 | 4.12 | -7.38 | -6.05 | -13.35 | 3 | 1 | -0.90 | 3.13 | 3.08 | 4.50 | 6.36 | 4.01 | 2.76 |

1 – code describing the acid-base properties: (-1) - acids; (0) - neutrals; (1) - bases

2 – multiplied by 10

3 – divided by 100

4 – divided by 10

**Table S3.** Chromatographic data and their derivatives obtained from NP TLC and RP TLC experiments

| API | $C_{NP}$* | NP** | NP/C | NP/PSA | $C_{RP}$* | RP** | RP/C | RP/PSA | NP/B2 | RP/B2 | NP/PB | RP/PB | NP/logP | RP/logP |
|---|---|---|---|---|---|---|---|---|---|---|---|---|---|---|
| **acetazolamid** | 0.94 | 0.94 | 1.00 | 0.817 | 0.97 | 0.96 | 0.99 | 0.834 | -0.727 | -0.742 | 0.959 | 0.980 | -3.615 | -3.692 |
| **amitriptyline** | 0.32 | 0.31 | 0.97 | 9.568 | 0.67 | 0.63 | 0.94 | 19.444 | 0.626 | 1.272 | 0.326 | 0.663 | 0.070 | 0.143 |
| **bupivacaine** | 0.67 | 0.67 | 1.00 | 2.072 | 0.74 | 0.75 | 1.01 | 2.319 | 22.666 | 25.372 | 0.705 | 0.789 | 0.202 | 0.227 |
| **chloroquine** | 0.17 | 0.17 | 1.00 | 0.604 | 0.40 | 0.30 | 0.75 | 1.065 | 1.763 | 3.111 | 0.309 | 0.545 | 0.039 | 0.068 |
| **citalopram** | 0.30 | 0.29 | 0.97 | 0.800 | 0.62 | 0.60 | 0.97 | 1.655 | -8.745 | -18.094 | 0.363 | 0.750 | 0.083 | 0.172 |
| **clomipramine** | 0.33 | 0.32 | 0.97 | 4.938 | 0.59 | 0.59 | 1.00 | 9.105 | 0.722 | 1.331 | 0.327 | 0.602 | 0.065 | 0.119 |
| **colchicine** | 0.79 | 0.77 | 0.97 | 0.927 | 0.91 | 0.91 | 1.00 | 1.095 | -0.984 | -1.163 | 1.974 | 2.333 | 0.720 | 0.850 |
| **diltiazem** | 0.49 | 0.49 | 1.00 | 0.829 | 0.76 | 0.72 | 0.95 | 1.219 | -1.230 | -1.808 | 0.653 | 0.960 | 0.104 | 0.152 |
| **doxepin** | 0.30 | 0.31 | 1.03 | 2.486 | 0.58 | 0.54 | 0.93 | 4.330 | 0.892 | 1.554 | 0.411 | 0.715 | 0.081 | 0.141 |
| **duloxetine** | 0.32 | 0.33 | 1.03 | 1.552 | 0.57 | 0.53 | 0.93 | 2.493 | 1.595 | 2.562 | 0.367 | 0.589 | 0.069 | 0.110 |
| **eletriptan** | 0.29 | 0.28 | 0.97 | 0.527 | 0.62 | 0.62 | 1.00 | 1.166 | -0.922 | -2.041 | 0.329 | 0.729 | 0.094 | 0.208 |
| **ethambutol** | 0.19 | 0.21 | 1.11 | 0.325 | 0.97 | 0.95 | 0.98 | 1.472 | -0.433 | -1.957 | 0.840 | 3.800 | -0.724 | -3.276 |
| **fluvoxamine** | 0.34 | 0.32 | 0.94 | 0.563 | 0.62 | 0.62 | 1.00 | 1.091 | -0.883 | -1.711 | 0.408 | 0.790 | 0.086 | 0.167 |
| **gabapentin** | 0.91 | 0.91 | 1.00 | 1.437 | 0.66 | 0.66 | 1.00 | 1.042 | -1.952 | -1.416 | 30.333 | 22.000 | 0.843 | 0.611 |
| **gentamycin** | 0.96 | 0.94 | 0.98 | 0.471 | 0.91 | 0.90 | 0.99 | 0.451 | -0.355 | -0.340 | 6.267 | 6.000 | -0.461 | -0.441 |
| **hydrochlorotiazide** | 0.96 | 0.96 | 1.00 | 0.811 | 0.97 | 0.96 | 0.99 | 0.811 | -0.713 | -0.713 | 1.414 | 1.414 | -48.000 | -48.000 |
| **itraconazole** | 0.96 | 0.97 | 1.01 | 0.962 | 0.86 | 0.84 | 0.98 | 0.833 | -0.910 | -0.788 | 0.972 | 0.842 | 0.214 | 0.185 |
| **ketorolac** | 0.92 | 0.91 | 0.99 | 1.535 | 0.96 | 0.96 | 1.00 | 1.619 | -2.265 | -2.389 | 0.919 | 0.970 | 0.340 | 0.358 |
| **levetiracetam** | 0.92 | 0.92 | 1.00 | 1.451 | 0.92 | 0.92 | 1.00 | 1.451 | -1.968 | -1.968 | 9.200 | 9.200 | -1.045 | -1.045 |
| **lincomycin** | 0.91 | 0.91 | 1.00 | 0.722 | 0.94 | 0.94 | 1.00 | 0.746 | -0.620 | -0.640 | 1.300 | 1.343 | 1.264 | 1.306 |
| **mesalazine** | 0.94 | 0.93 | 0.99 | 1.113 | 0.99 | 0.97 | 0.98 | 1.161 | -1.178 | -1.228 | 2.163 | 2.256 | 1.257 | 1.311 |
| **minoxidil** | 0.43 | 0.42 | 0.98 | 0.449 | 0.80 | 0.82 | 1.03 | 0.876 | -0.442 | -0.862 | | | 0.259 | 0.506 |
| **nitrendipine** | 0.97 | 0.97 | 1.00 | 0.878 | 0.86 | 0.86 | 1.00 | 0.779 | -0.795 | -0.705 | 0.980 | 0.869 | 0.255 | 0.226 |
| **ofloxacin** | 0.24 | 0.23 | 0.96 | 0.314 | 0.54 | 0.50 | 0.93 | 0.682 | -0.367 | -0.799 | 0.719 | 1.563 | 0.124 | 0.270 |
| **oxcarbamazepine** | 0.91 | 0.90 | 0.99 | 1.420 | 0.91 | 0.91 | 1.00 | 1.435 | -1.926 | -1.947 | 2.250 | 2.275 | 0.542 | 0.548 |
| **pefloxacin** | 0.30 | 0.29 | 0.97 | 0.452 | 0.76 | 0.70 | 0.92 | 1.092 | -0.606 | -1.463 | 1.160 | 2.800 | 0.151 | 0.365 |
| **pregabalin** | 0.76 | 0.76 | 1.00 | 1.200 | 0.41 | 0.41 | 1.00 | 0.648 | -1.630 | -0.880 | | | 0.697 | 0.376 |
| **primidone** | 0.90 | 0.93 | 1.03 | 1.598 | 0.93 | 0.97 | 1.04 | 1.667 | -2.421 | -2.525 | 1.329 | 1.386 | 1.120 | 1.169 |

| | | | | | | | | | | | | | | |
|---|---|---|---|---|---|---|---|---|---|---|---|---|---|---|
| **propylthiouracil** | 0.94 | 0.94 | 1.00 | 2.285 | 0.93 | 0.93 | 1.00 | 2.261 | -8.462 | -8.372 | 1.146 | 1.134 | 0.817 | 0.809 |
| **quinine** | 0.30 | 0.29 | 0.97 | 0.636 | 0.66 | 0.64 | 0.97 | 1.404 | -1.590 | -3.508 | 0.414 | 0.914 | 0.103 | 0.227 |
| **rifampicin** | 0.73 | 0.77 | 1.05 | 0.350 | 0.95 | 0.94 | 0.99 | 0.427 | -0.259 | -0.316 | 0.865 | 1.056 | 0.376 | 0.459 |
| **rimantadine** | 0.92 | 0.92 | 1.00 | 3.536 | 0.95 | 0.95 | 1.00 | 3.651 | 7.040 | 7.270 | 2.300 | 2.375 | 0.278 | 0.287 |
| **timolol** | 0.34 | 0.32 | 0.94 | 0.401 | 0.97 | 0.97 | 1.00 | 1.216 | -0.439 | -1.331 | 3.200 | 9.700 | 0.250 | 0.758 |
| **tinidazole** | 0.89 | 0.88 | 0.99 | 0.900 | 0.94 | 0.93 | 0.99 | 0.951 | -0.865 | -0.914 | 7.333 | 7.750 | -3.034 | -3.207 |
| **tramadol** | 0.31 | 0.31 | 1.00 | 0.948 | 0.63 | 0.64 | 1.02 | 1.957 | 13.025 | 26.891 | 1.550 | 3.200 | 0.134 | 0.276 |
| **trimethoprim** | 0.57 | 0.59 | 1.04 | 0.559 | 0.84 | 0.83 | 0.99 | 0.787 | -0.517 | -0.727 | 1.341 | 1.886 | 1.000 | 1.407 |
| **warfarin** | 0.99 | 0.99 | 1.00 | 1.557 | 0.87 | 0.83 | 0.95 | 1.305 | -2.104 | -1.764 | 1.000 | 0.838 | 0.316 | 0.265 |

* Rf value from unimpregnated NP or RP plate (control)

** Rf value from NP or RP plate impregnated with 2 mg/mL BSA solution

**Table S4.** Chromatographic data and their derivatives obtained from the $HPLC_{HSA}$ i $HPLC_{IAM}$ experiments

| API | $k_{HSA}$ | log $k_{HSA}$ | log $k_{HSA}$/B2 | log $k_{HSA}$/PB | log $k_{HSA}$/log P | $k_{IAM}$ | log $k_{IAM}$ |
|---|---|---|---|---|---|---|---|
| **acetazolamid** | 0.74 | -0.13 | -0.575 | 0.760 | -2.863 | 2.90 | 0.46 |
| **amitriptyline** | 5.87 | 0.77 | 11.854 | 6.178 | 1.331 | 1.56 | 0.19 |
| **bupivacaine** | 1.18 | 0.07 | 40.084 | 1.247 | 0.358 | 3.02 | 0.48 |
| **chloroquine** | 18.05 | 1.26 | 187.127 | 32.812 | 4.092 | 3.11 | 0.49 |
| **citalopram** | 2.67 | 0.43 | -80.418 | 3.333 | 0.766 | 3.14 | 0.50 |
| **clomipramine** | 9.09 | 0.96 | 20.510 | 9.278 | 1.841 | 3.05 | 0.48 |
| **colchicine** | 0.28 | -0.55 | -0.362 | 0.726 | 0.265 | 3.61 | 0.56 |
| **diltiazem** | 1.74 | 0.24 | -4.362 | 2.316 | 0.367 | 3.96 | 0.60 |
| **doxepin** | 4.25 | 0.63 | 12.220 | 5.624 | 1.106 | 2.81 | 0.45 |
| **duloxetine** | 8.16 | 0.91 | 39.435 | 9.063 | 1.696 | 2.84 | 0.45 |
| **eletriptan** | 3.78 | 0.58 | -12.429 | 4.441 | 1.267 | 3.57 | 0.55 |
| **ethambutol** | 1.73 | 0.24 | -3.557 | 6.906 | -5.953 | 2.18 | 0.34 |
| **fluvoxamine** | 0.25 | -0.60 | -0.696 | 0.321 | 0.068 | 2.79 | 0.45 |
| **gabapentin** | 0.15 | -0.84 | -0.312 | 4.843 | 0.135 | 1.70 | 0.23 |
| **gentamycin** | 1.50 | 0.18 | -0.568 | 10.028 | -0.737 | 4.25 | 0.63 |
| **hydrochlorotiazide** | 0.61 | -0.22 | -0.452 | 0.897 | -30.451 | 2.01 | 0.30 |
| **itraconazole** | 9.65 | 0.98 | -9.055 | 9.668 | 2.130 | 6.15 | 0.79 |
| **ketorolac** | 9.65 | 0.98 | -24.017 | 9.747 | 3.601 | 2.39 | 0.38 |
| **levetiracetam** | 0.07 | -1.16 | -0.149 | 0.696 | -0.079 | 1.62 | 0.21 |
| **lincomycin** | 0.82 | -0.08 | -0.560 | 1.175 | 1.142 | 3.89 | 0.59 |
| **mesalazine** | 0.18 | -0.73 | -0.234 | 0.430 | 0.250 | 1.28 | 0.11 |
| **minoxidil** | 0.40 | -0.40 | -0.422 | | 0.248 | 1.96 | 0.29 |
| **nitrendipine** | 3.55 | 0.55 | -2.906 | 3.581 | 0.931 | 2.98 | 0.47 |
| **ofloxacin** | 6.62 | 0.82 | -10.566 | 20.673 | 3.576 | 3.07 | 0.49 |
| **oxcarbamazepine** | 0.40 | -0.40 | -0.846 | 0.988 | 0.238 | 2.26 | 0.35 |
| **pefloxacin** | 12.43 | 1.09 | -25.980 | 49.720 | 6.474 | 2.91 | 0.46 |
| **pregabalin** | 0.14 | -0.87 | -0.291 | | 0.124 | 1.65 | 0.22 |
| **primidone** | 0.22 | -0.66 | -0.569 | 0.312 | 0.263 | 2.02 | 0.31 |

| | | | | | | | |
|---|---|---|---|---|---|---|---|
| **propylthiouracil** | 0.33 | -0.48 | -2.950 | 0.400 | 0.285 | 1.51 | 0.18 |
| **quinine** | 2.59 | 0.41 | -14.194 | 3.699 | 0.918 | 3.06 | 0.49 |
| **rifampicin** | 0.03 | -1.53 | -0.010 | 0.034 | 0.015 | 8.37 | 0.92 |
| **rimantadine** | 0.16 | -0.80 | 1.222 | 0.399 | 0.048 | 1.91 | 0.28 |
| **timolol** | 0.13 | -0.88 | -0.179 | 1.304 | 0.102 | 2.88 | 0.46 |
| **tinidazole** | 0.12 | -0.92 | -0.118 | 0.997 | -0.413 | 2.00 | 0.30 |
| **tramadol** | 1.08 | 0.03 | 45.221 | 5.381 | 0.464 | 3.90 | 0.59 |
| **trimethoprim** | 0.85 | -0.07 | -0.749 | 1.943 | 1.449 | 2.63 | 0.42 |
| **warfarin** | 13.86 | 1.14 | -29.454 | 14.001 | 4.428 | 2.76 | 0.44 |

**Table S5.** Structures of 37 APIs

| 1. acetazolamid | 2. amitriptyline | 3. bupivacaine | 4. chloroquine |
|---|---|---|---|
| 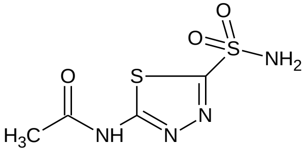 | 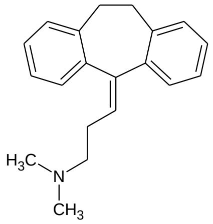 | 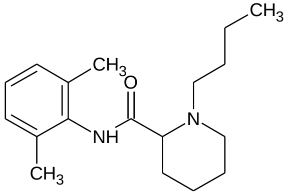 | 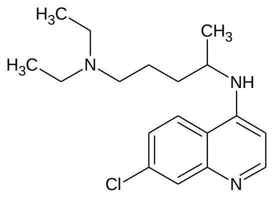 |
| 5. citalopram | 6. clomipramine | 7. colchicine | 8. diltiazem |
| 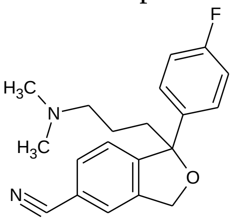 | 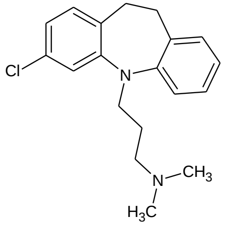 | 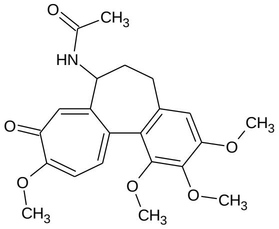 | 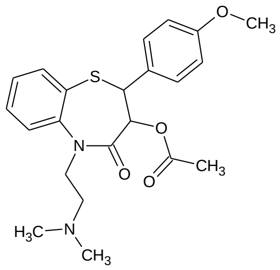 |
| 9. doxepin | 10. duloxetine | 11. eletriptan | 12. ethambutol |
| 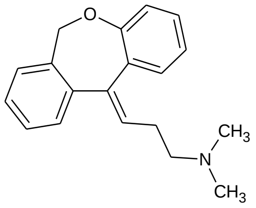 | 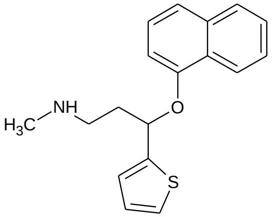 | 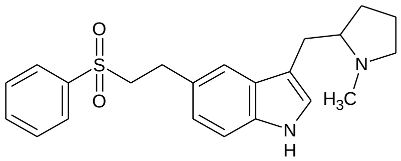 | 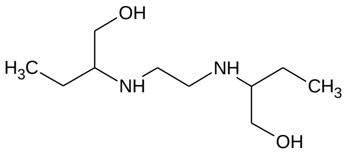 |

13. fluvoxamine

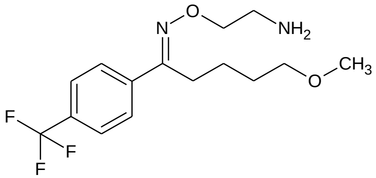

14. gabapentin

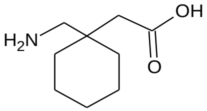

15. gentamicin

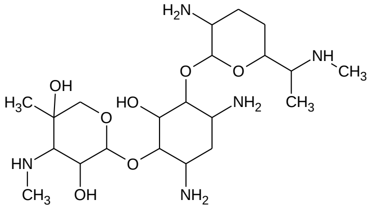

16. hydrochlorotiazide

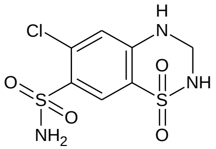

17. itraconazole

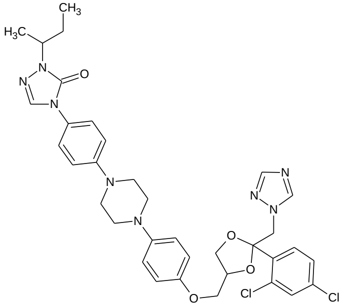

18. ketorolac

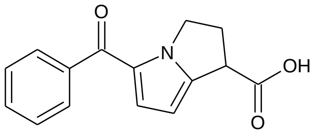

19. levetiracetam

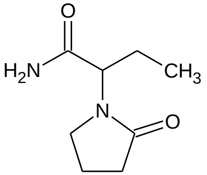

20. lincomycin

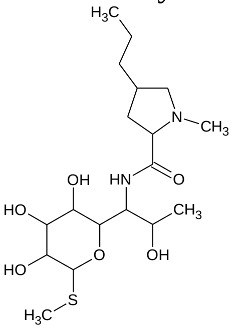

21. mesalazine

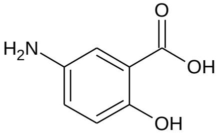

22. minoxidil

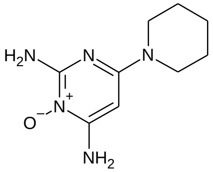

23. nitrendipine

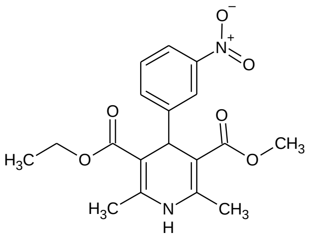

24. ofloxacin

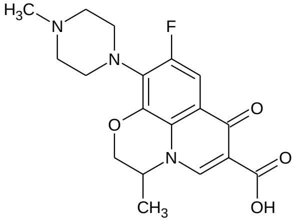

25. oxcarbamazepine

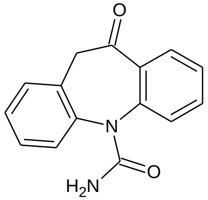

26. pefloxacin

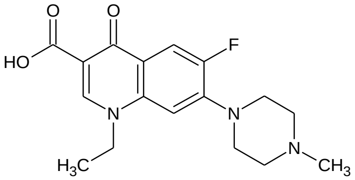

27. pregabalin

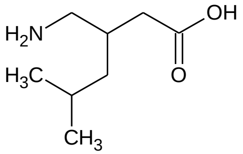

28. primidone

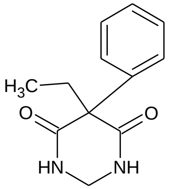

29. propylthiouracil

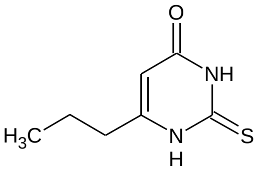

30. quinine

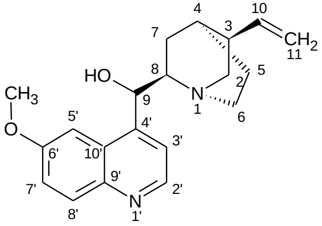

31. rifampicin

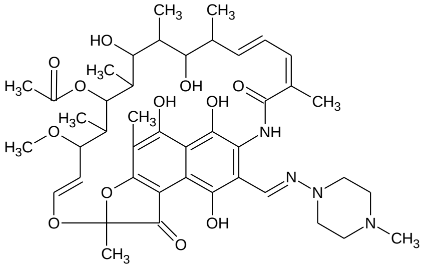

32. rimantadine

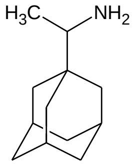

33. timolol

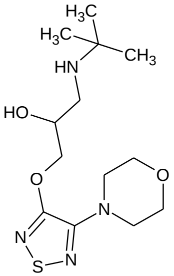

34. tinidazole

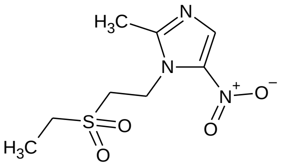

35. tramadol

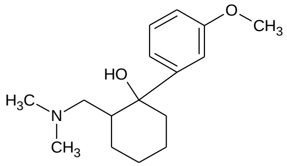

36. trimethoprim

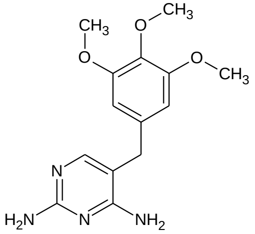

37. warfarin

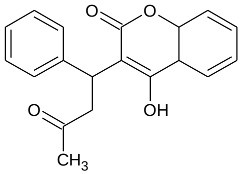

Supplement: Supplementary file 1 [file cells-12-00421-s001.zip › cells-2099421-supplementary.pdf]
